# Iguratimod Inhibits the Aggressiveness of Rheumatoid Fibroblast-Like Synoviocytes

**DOI:** 10.1155/2019/6929286

**Published:** 2019-11-14

**Authors:** Jin Lin, Ye Yu, Xuanwei Wang, Yini Ke, Chuanyin Sun, Lihuan Yue, Guanhua Xu, Bei Xu, Liqin Xu, Heng Cao, Danyi Xu, Nancy Olsen, Weiqian Chen

**Affiliations:** ^1^Department of Rheumatology, the First Affiliated Hospital, College of Medicine, Zhejiang University, Hangzhou, 310003 Zhejiang Province, China; ^2^Department of Orthopedics, the First Affiliated Hospital, College of Medicine, Zhejiang University, Hangzhou, 310003 Zhejiang Province, China; ^3^Division of Rheumatology, Department of Medicine, Penn State University Hershey College of Medicine, Hershey 17033, USA

## Abstract

**Objective:**

Iguratimod, a novel disease-modifying anti-rheumatic drug for the treatment of rheumatoid arthritis, has been approved in China and Japan. Here, we aimed to find whether iguratimod can inhibit the aggressive behavior and promote apoptosis of rheumatoid fibroblast-like synoviocytes (RA-FLSs).

**Methods:**

The proliferation of RA-FLSs was assessed by 5-ethynyl-2′-deoxyuridine test and Cell Counting Kit-8. Migration and invasion were determined by the wound test and a transwell assay. Apoptosis was tested by flow cytometry. The mRNA expression of matrix metalloproteinases (MMPs) and proinflammatory cytokines in RA-FLSs were measured by quantitative PCR and ELISA. To gain insight into the molecular signaling mechanisms, we determined the effect of iguratimod on the activation of mitogen-activated protein kinases (MAPK) signaling pathways by the cellular thermal shift assay (CETSA) and western blot.

**Results:**

Iguratimod treatment significantly reduced the proliferation, migration, and invasive capacities of RA-FLSs in a dose-dependent manner *in vitro*. MMP-1, MMP-3, MMP-9, Interleukin-6 (IL-6), and monocyte chemoattractant protein-1 mRNA and protein levels were all decreased after treatment with iguratimod. Furthermore, tumor necrosis factor-alpha- (TNF-*α*-) induced expression of phosphorylated c-Jun N-terminal kinases (JNK) and P38 MAPK were inhibited by iguratimod. Additionally, iguratimod promoted the apoptosis of RA-FLSs. Most importantly, iguratimod was shown to directly interact with JNK and P38 protein by CETSA assay. Moreover, activating transcription factor 2 (ATF-2), a substrate of both JNK and P38, was suppressed by iguratimod.

**Conclusions:**

Our findings suggested that the therapeutic effects of iguratimod on RA might be, in part, due to targeting the aggressive behavior and apoptosis of RA-FLSs.

## 1. Introduction

Rheumatoid arthritis (RA) is a chronic autoimmune disorder characterized by joint involvement and systemic features which affects 0.28% of Chinese people [1]. It can lead to significant morbidity and mortality. However, the pathogenesis and etiology of RA remain unclear. Solid evidence supports that activated fibroblast-like synoviocytes (FLSs) are key effector cells in the pathophysiological course of RA [2]. As we know, RA-FLSs contribute to the production of inflammatory cytokines and matrix metalloproteinases (MMPs) that degrade the extracellular matrix. Moreover, RA-FLSs display a highly proliferative and invasive behavior which is similar to tumor cells and is critical in the development of pannus by migrating and invading the cartilage and bone [3]. RA-FLSs have an aggressive capacity to invade the extracellular matrix, mediating inflammation and further exacerbating joint damage. RA-FLSs cultured *in vitro* still retain their aggressive phenotype in the absence of exogenous stimulation. The strongest evidence to support that RA-FLSs not only invade and degrade human cartilage when coimplanted with the tissue into mice with severe combined immunodeficiency (SCID) is that these cells also migrate to the contralateral implanted human cartilage that had been inserted without RA-FLSs and erode the cartilage. Thus, RA-FLSs play a crucial role in invasive synovium and initiation and perpetuation of destructive joint inflammation. Importantly, RA-FLSs are protected from apoptosis due to strong survival signaling [2]. Therefore, targeting RA-FLSs to modulate FLSs invasiveness or apoptosis may be a novel therapeutic approach for RA.

Proper treatment is essential in decreasing the burden of RA. Iguratimod is a novel disease-modifying antirheumatic drug that has been approved for the treatment of RA in China and Japan [4]. Initially, iguratimod was classified as a nonsteroidal anti-inflammatory drug (NSAID). Subsequently, it was demonstrated that iguratimod not only suppressed T cell proliferation and production of interferon- (IFN-) *γ*, Interleukin- (IL-) 1*β*, IL-6, IL-17, and tumor necrosis factor-alpha (TNF-*α*), in T cells but also inhibited the production of IgM and IgG by B cells [4–8]. In the CIA rat model, iguratimod was proved to be an effective disease-modifying agent that can prevent bone/cartilage destruction and inflammation [8]. Clinically, iguratimod is comparable to methotrexate (MTX) or sulfasalazine for RA in terms of American College of Rheumatology (ACR) 20% improvement criteria response rate in a multicenter, randomized, double-blind, controlled trial [9]. Furthermore, side effects in the iguratimod group were generally fewer and milder than those in the MTX or sulfasalazine group [10]. Moreover, the combination with iguratimod and MTX markedly enhanced the therapeutic effect of iguratimod [8]. Importantly, iguratimod in combination with MTX was efficacious with a manageable safety profile in patients with active RA who had an inadequate response to stable background MTX alone [11]. Collectively, iguratimod was a useful agent to treat patients with RA.

It was demonstrated that serum MMP-1 and MMP-3 were decreased significantly in patients with RA after 24 weeks treatment of iguratimod [12, 13]. Furthermore, iguratimod could reduce murine arthritis through targeting Act1-mediated IL-17 signaling [4]. However, the role of iguratimod in regulating biological properties of RA-FLSs is unclear. In our study, we explored whether iguratimod could affect invasion features and apoptosis of RA-FLSs.

## 2. Materials and Methods

### 2.1. Chemicals

Iguratimod was kindly provided by Simcere Pharmaceutical (Nanjing, China). Human recombinant TNF-*α* and platelet-derived growth factor (PDGF) were purchased from R&D Systems. Rabbit polyclonal or mouse monoclonal Ab against phospho- or total c-Jun N-terminal kinases (JNK), P38 mitogen-activated protein kinases (MAPK), extracellular signal-regulated kinases (ERK), activating transcription factor 2 (ATF-2), and ELK-1 were all purchased from Cell Signaling Technology (Danvers, MA, USA). A mouse antibody (Ab) against glyceraldehyde-3-phosphate dehydrogenase (GAPDH) was purchased from LianKe Company (Hangzhou, China).

### 2.2. Preparation and Culture of RA-FLSs

Synovial tissue specimens were collected from 8 female patients with active RA who all fulfilled the ACR 1987 revised criteria for the classification or 2010 ACR/European League Against Rheumatism (EULAR) criteria of RA [14, 15] and who underwent synovectomy or joint replacement surgery. All of the patients provided written informed consent before the procedure. The study was performed according to the recommendations of the Declaration of Helsinki and was approved by the Medical Ethical Committee (number: 2017499) of the First Affiliated Hospital, Zhejiang University, China. RA synovial fibroblasts were isolated from the synovial tissue of each RA patient separately. The synovial tissue was finely minced and digested with 1 mg/ml type I collagenase (Sigma-Aldrich, USA) in serum-free Dulbecco's Modified Eagle Medium (DMEM) medium at 37°C, 5% CO_2_ for 2 hours (h). The cell suspension was filtered through a sterile cell strainer (BD Biosciences); synoviocytes were collected and rinsed by centrifugation. The pellet was resuspended in DMEM containing 10% fetal bovine serum (FBS), 100 U/ml penicillin, and 100 *μ*g/ml streptomycin, was transferred to a 100 mm dish, and incubated at 37°C, 5% CO_2_. When cells reached more than 90% confluency, RA-FLSs were trypsinized and passaged. The RA-FLSs obtained from the 4th to 6th passages were used for experiments.

To determine the effect of iguratimod on the biological function of RA-FLSs, different concentrations were added into the culture system in the experiments described below.

### 2.3. 5-Ethynyl-2′-Deoxyuridine (Edu) Tests

RA-FLSs were cultured in a serum-free medium for 24 h at a density of 1 × 10^4^ cells/well in 48-well plates. After serum starvation, the cells were incubated with PDGF (10 ng/ml) for 72 h and then incubated with 50 *μ*M EdU for 2 h. Then, cells were fixed with paraformaldehyde, stained by Apollo fluid and Hoechst33342. Edu incorporation was assessed using a fluorescence microscope according to manufacturer's instructions (RiboBio Co., Ltd., Guangzhou, China).

### 2.4. Cell Counting Kit-8 (CCK-8)

RA-FLSs were incubated in a 96-well plate. Preincubation of the plate was in a humidified incubator 37°C, 5% CO_2_, followed by addition of 10 *μ*l of the CCK-8 solution to each well of the plate. Incubation of the plate was for 1-4 h in the incubator. Measured absorbance at 450 nm was done using a microplate reader. A calibration curve was prepared using the data obtained from the wells that contain known numbers of viable cells.

### 2.5. Wound Healing Assay

In the wound healing assay, RA-FLSs were cultured to 80–90% confluence in 24-well plates. The cell monolayer was scratched in a straight line with a 200 *μ*l sterile pipette tip and was washed with phosphate-buffered saline (PBS) to remove the floating cells. The RA-FLSs were cultured in the presence of 2% FBS with or without treatment with iguratimod at various concentrations for 48 h. The speed of wound closure was analyzed by measuring the cells that moved from the wound edge to the center after 48 h.

### 2.6. Transwell Migration/Invasion Assay

RA-FLSs were starved in serum-free DMEM for 24 h, and a transwell assay was then performed using 6.5 mm transwell chambers with 8 *μ*m pores. Briefly, the bottom surface of each membrane was precoated with Matrigel. Then, RA-FLSs (1 × 10^4^, 200 *μ*l) were seeded in the upper chambers and 600 *μ*l of the complete medium contained 10% FBS was added to the lower chambers. After incubation at 37°C for 24 h, the upper surface of each membrane was cleaned with a cotton swab. Cells that migrated to the bottom side were stained with crystal violet and counted under a microscope.

### 2.7. Western Blot Analysis

Protein lysates obtained from equal numbers of RA-FLSs were subjected to sodium dodecyl sulfate-polyacrylamide gel electrophoresis (SDS-PAGE) using standard electrophoresis and transfer techniques as previously described [16]. Equal amounts of protein were loaded. Membranes were incubated with antibodies against total JNK, P38(D13E1), ERK1/2(137F5), ATF-2(D4L2X), ELK-1, and phospho-JNK(pT183/pY185, 81E11), phospho-P38(Thr180/Tyr182, D3F9), phospho-ERK(Thr202/Tyr204), and phospho-ATF-2 (Thr69/71) as well as GAPDH antibody overnight at 4°C, followed by incubation with secondary antibody-HRP conjugate for 1 h at room temperature. GAPDH was used as a protein loading control. Immunoreactive bands were detected using enhanced chemiluminescence and autoradiography.

### 2.8. RNA Extraction, cDNA Synthesis, and Semiquantitative Real-Time PCR

RA-FLSs were preserved in TRIzol reagent (Invitrogen, Shanghai, China) and stored at -80°C. Total RNA was extracted. cDNA was prepared from 1 *μ*g of RNA using the PrimeScript Reverse Transcriptase kit with gDNA eraser (Takara, Japan). The resulting cDNA was amplified by real-time PCR using a 7900HT Fast Real-time PCR thermal cycler. Amplification was performed using SYBR Green (Takara, Japan). The primers for the target gene are described below:

MMP-1, forward: 5′-GCTAACAAATACTGGAGGTATGATG-3′, reverse: 5′-ATTTTGGGATAACCTGGATCCATAG-3′; MMP-3, forward: 5′-AGCAAGGACCTCGTTTTCATT-3′, reverse: 5′-GTCAATCCCTGGAAAGTCTTCA-3′; MMP-9, forward: 5′-AGACCTGGGCAGATTCCAAAC-3′, reverse: 5′-CGGCAAGTCTTCCGAGTAGT-3′; IL-6, forward: 5′-ACTCACCTCTTCAGAACGAATTG-3′, reverse: 5′-CCATCTTTGGAAGGTTCAGGTTG-3′; MCP-1, forward: 5′-CAGCCAGATGCAATCAATGCC-3′, reverse: 5′-TGGAATCCTGAACCCACTTCT-3′; Caspase 3, forward: 5′-CATGGAAGCGAATCAATGGACT-3′, reverse: 5′-CTGTACCAGACCGAGATGTCA-3′; cFLIP, forward: 5′-TGCTCTTTTTGTGCCGGGAT-3′, reverse: 5′-CGACAGACAGCTTACCTCTTTC-3′; GAPDH, forward: 5′-GGAGCGAGATCCCTCCAAAAT-3′, reverse: 5′-GGCTGTTGTCATACTTCTCATGG-3′.

GAPDH were considered a normalization control. The data were examined using the 2^-*ΔΔ*CT^ method, and results were expressed as fold increase. Each sample was tested in triplicate, and the tests were repeated three times.

### 2.9. Apoptosis Assays

RA-FLSs were cultured with different concentrations of iguratimod in the presence or absence of TNF-*α* (25 ng/ml) for 24 hours. The cells were then collected, stained with CD90 (5E10, BioLegend, CA, USA), Annexin V, and propidium iodide (PI) using an Annexin V apoptosis detection kit (BD Biosciences, USA) following manufacturer's instructions. Both Annexin V and PI expression were measured by FACSCalibur flow cytometer or Accuri C6 Cytometer (BD Biosciences, USA) gated on CD90^+^ RA-FLSs.

### 2.10. Cellular Thermal Shift Assay (CETSA) by Immunoblotting

The CETSA method was modified from a published protocol [17]. Briefly, RA-FLSs cells were seeded in 10 cm plates and grown to 80%-90% confluence. Cells were treated with either 5 *μ*g/ml iguratimod or dimethyl sulfoxide (DMSO) for 1 h. Following treatment, the cells were trypsinized, pelleted, and washed 2 times with PBS. Each sample was suspended in equivalent PBS supplemented with protease inhibitors after they were washed in PBS. And then, these samples were heated at 41, 46, 49, 55, 58, and 61°C for 3 min. Immediately after heating, tubes were removed and incubated at room temperature for 3 min, and then snap-frozen in liquid nitrogen for 3 min. Samples were freeze-thawed at 25°C for 3 min, then centrifuged at 20,000*g* for 20 min at +4°C. The supernatants were analyzed by western blotting.

### 2.11. Statistical Analysis

Data were expressed as mean ± SD unless otherwise indicated. Data were analyzed using one-way ANOVA followed by Turkey's test. Differences were considered statistically significant when *p* < 0.05. All analyses were performed using the statistical package SPSS 18.0 (SPSS, Chicago, IL).

## 3. Results

### 3.1. Iguratimod Suppressed Proliferation of RA-FLSs in a Dose-Dependent Manner

**Figure 1:**
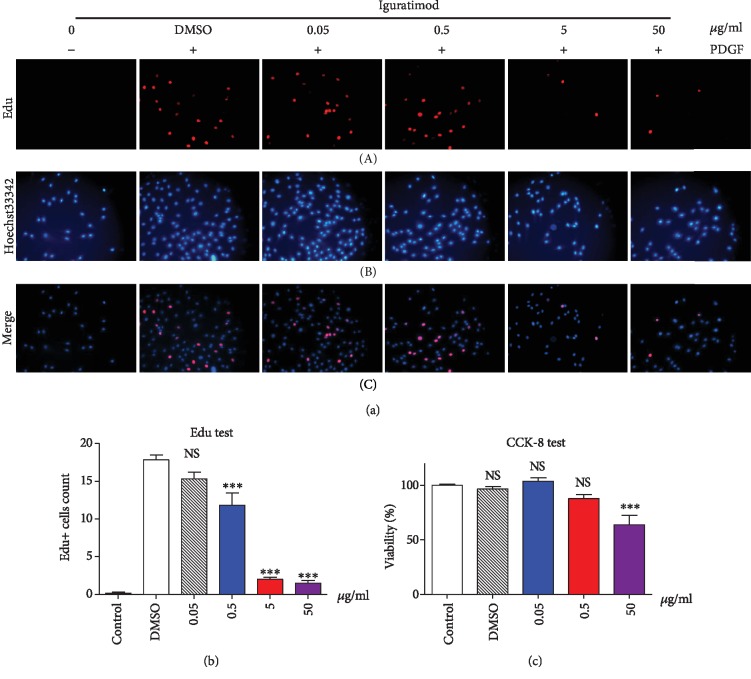
Iguratimod suppressed proliferation of RA-FLSs in a dose-dependent manner. RA-FLSs were starved for 24 h and then cultured in the presence of platelet-derived growth factor (PDGF) (10 ng/ml) with or without treatment with iguratimod at various concentrations for 72 h. Immunofluorescence staining for 5-ethynyl-2-deoxyuridine (Edu) (red, A), Hoechst 33342 (blue, B), and merged images (C) in the nucleus of cells were shown. Representative pictures (a) and the summarized data (b) of three separate experiments were shown. (c) The proliferation rate of RA-FLSs was also measured by the CCK-8 method. The data indicated the mean ± SEM of 3 separate experiments (totally 6 RA-FLS lines). The data were analyzed using one-way ANOVA followed by Turkey's test. ^∗∗∗^*P* < 0.001 versus the DMSO group. NS: not significant.

To assess the influence of iguratimod on the proliferation of RA-FLSs, RA-FLSs were stimulated by PDGF and incubated with different doses of iguratimod (0, 0.05, 0.5, 5, and 50 *μ*g/ml), and the proliferation was assessed by Edu incorporation assay. We found that iguratimod suppressed proliferation of RA-FLSs in a dose-dependent manner (Figures 1(a) and 1(b)). Meanwhile, CCK-8 experiments showed similar results (Figure 1(c)), further to confirm the suppression of Iguratimod on the proliferation of RA-FLSs.

### 3.2. Iguratimod Inhibited MMPs and Inflammatory Cytokine Production of RA-FLSs

**Figure 2:**
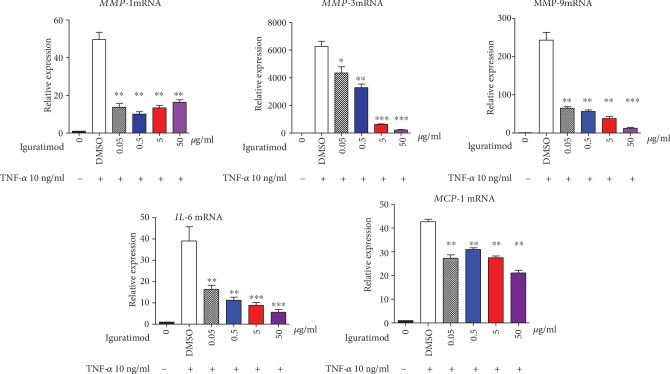
Iguratimod inhibited mRNA expressions of MMPs and inflammatory cytokines of RA-FLSs. RA-FLSs were stimulated with TNF-*α* 10 ng/ml and different concentrations of iguratimod or DMSO for 4 h, then cells were collected; mRNAs were extracted; MMP-1, MMP-3, MMP-9, IL-6, and MCP-1 mRNA expressions were measured by qPCR. The data were shown as the mean ± SD for three independent experiments, each in triplicate. The data were analyzed using one-way ANOVA followed by Turkey's test. ^∗^*P* < 0.05, ^∗∗^*P* < 0.01, ^∗∗∗^*P* < 0.001 versus the DMSO group.

**Figure 3:**
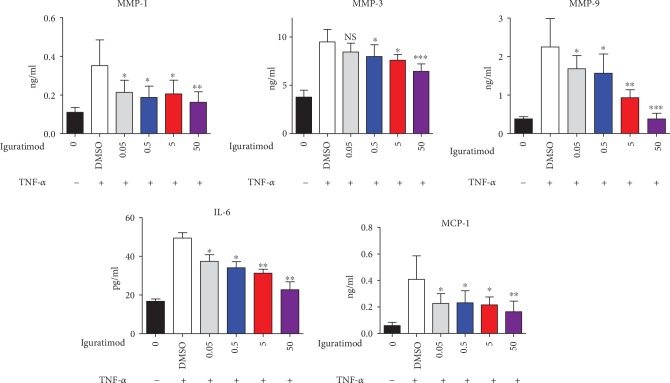
Iguratimod inhibited protein levels of MMPs and inflammatory cytokines of RA-FLSs. RA-FLSs were stimulated with TNF-*α* 10 ng/ml and different concentrations of iguratimod or DMSO for 4 h, then supernatants were collected, MMP-1, MMP-3, MMP-9, IL-6, and MCP-1 protein levels were measured by ELISA. The data were shown as the mean ± SD for three independent experiments. The data were analyzed using one-way ANOVA followed by Turkey's test. ^∗^*P* < 0.05 versus the DMSO group, ^∗∗^*P* < 0.01 versus the DMSO group, ^∗∗∗^*P* < 0.001 versus the DMSO group.

High expression of MMPs and inflammatory cytokines was a hallmark characteristic of RA-FLSs. Therefore, we checked the role of iguratimod in the regulation of MMPs and inflammatory cytokine production by TNF-*α* stimulated RA-FLSs. As shown in Figure 2, MMP-1, MMP-3, MMP-9, IL-6, and MCP-1 mRNA were all decreased in the iguratimod-treated group, when compared to the DMSO group. Interestingly, MMP-3, MMP-9, and IL-6 were much lower in the high-dose group. We also did the ELISA tests for MMPs and inflammatory cytokines in the supernatant. This represented protein levels of RA-FLSs before and after treatment with iguratimod. We found consistent results showing that iguratimod reduced the protein levels of MMP-1, MMP-3, MMP-9, IL-6, and MCP-1 (Figure 3).

### 3.3. Iguratimod Suppressed the Migration and Invasion of RA-FLSs

**Figure 4:**
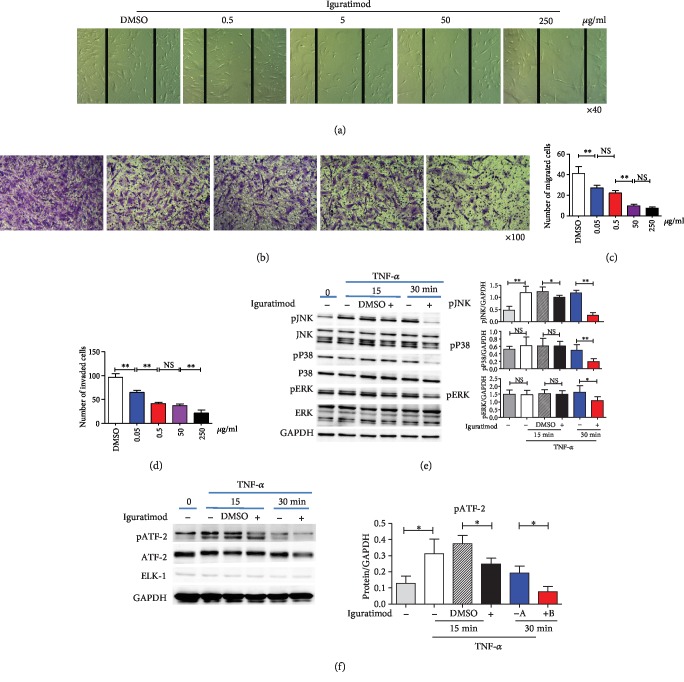
Iguratimod suppressed the migration and invasion of RA-FLSs by blocking MAPK signaling. (a, c) RA-FLS monolayers were scratched with a sterile pipette tip. Each well was treated with 2% FBS and iguratimod at various concentrations. Microscopy images were acquired at 48 h after wounds were created. Representative microscopy images from the wound assay were shown. (b, d) RA-FLSs were starved in serum-free DMEM for 24 h, FLSs (1 × 10^4^, 200 *μ*l) were seeded in the upper chambers, treated with different concentrations of iguratimod for 24 h, and then a transwell assay was performed. The data shown were the mean ± SD for three independent experiments (totally 6 RA-FLSs lines). (e) RA-FLSs were cultured in the presence of TNF-*α* (10 ng/ml) with or without iguratimod or DMSO at indicated times. Protein lysates obtained from equal numbers of RA-FLSs were analyzed by western blot. Total JNK, P38, ERK, phospho-JNK, phospho-P38, and phospho-ERK, as well as GAPDH, were detected. (f) RA-FLSs were cultured in the presence of TNF-*α* (10 ng/ml) with or without iguratimod or DMSO at indicated times. Protein lysates obtained from equal numbers of RA-FLSs were analyzed by western blot. Protein pATF-2, total ATF-2, ELK-1, and GAPDH were detected. The experiments were repeated four times (totally 4 RA-FLSs lines). The representative plots were shown. GAPDH was used as endogenous control, and relative expression of each protein is shown as a protein/GAPDH ratio. The summarized data were shown. The experiments were repeated three times. The data were analyzed using one-way ANOVA followed by Turkey's test. ^∗^*P* < 0.05, ^∗∗^*P* < 0.01 versus the control group.

Next, we investigated the impact of iguratimod on migration and invasion of RA-FLSs. RA-FLSs were treated with iguratimod, and a wound healing assay was conducted to measure the effects on migration. As shown in Figures 4(a) and 4(c), cells treated with iguratimod were less likely to migrate into the created cell-free area than the DMSO control group. Higher concentrations of iguratimod displayed stronger suppression. Furthermore, iguratimod markedly inhibited the FBS-induced migration and invasion of RA-FLSs in a dose-dependent manner by a transwell chamber assay with Matrigel (Figures 4(b) and 4(d)), indicating that iguratimod suppressed migration and invasion of RA-FLSs.

### 3.4. Effect of Iguratimod on the MAPKs Signaling in RA-FLSs

Since MAPK signaling pathways are crucial for FLS migration, invasion, proliferation, and inflammatory cytokine production [18], we next investigated whether iguratimod had any effect on MAPK (JNK, P38, and ERK) activation in response to TNF-*α*. As shown in Figure 4(e), phosphorylation of JNK was clearly upregulated after treatment with TNF-*α*. Phosphorylation of P38 and ERK were found at RA-FLSs without TNF-*α* stimulation. We demonstrated that iguratimod treatment suppressed the phosphorylation of JNK and P38 after 30 minutes of stimulation. Meanwhile, iguratimod had only slightly decreased the effect on pERK activation after 30 minutes. It had been reported the ATF-2 and ELK-1 are two transcription factors and substrates of both JNK and P38 signaling pathway [19, 20]. Most importantly, ATF-2 can be induced by TNF-*α* in RA FLS, contributing to the pathogenesis of RA [21]. Next, we tested whether iguratimod had any effects on ATF-2 and ELK-1 expression. We revealed that phosphorylation of ATF-2, not ELK-1, was clearly upregulated after treatment with TNF-*α* (Figure 4(f)). We also demonstrated that iguratimod treatment suppressed the pATF-2 expression after 15 and 30 minutes of stimulation.

### 3.5. Iguratimod Promoted Apoptosis of RA-FLSs

**Figure 5:**
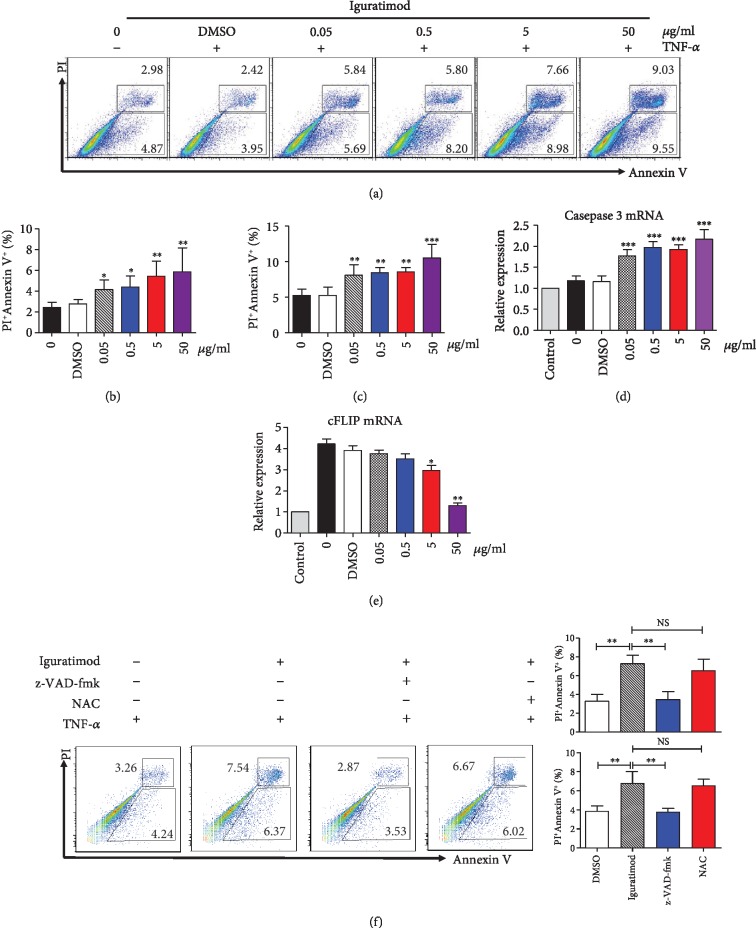
Iguratimod promoted apoptosis of RA-FLSs. (a) RA-FLSs were treated with TNF-*α* (25 ng/ml) and different concentrations of iguratimod for 24 h. Propidium iodide (PI) and Annexin V (AV) staining were determined by flow cytometry. Typical flow plots were shown. (b, c) The summary data of early apoptotic cells (PI^−^AV^+^) and late apoptotic or dead cells (PI^+^AV^+^) were shown. The data were described as the mean ± SD for three independent experiments (totally 8 RA-FLSs lines). (d, e) Caspase 3 and cFLIP mRNA expression were measured by qPCR in RA-FLSs. (f) For inhibitor experiments, cells were pretreated with the pan-caspase inhibitor z-VAD-fmk (Sigma-Aldrich, 10 *μ*M) or N-acetyl-l-cysteine (NAC, Sigma-Aldrich, 5 mM) for 1 h before the treatment of iguratimod. RA-FLSs were treated with TNF-*α* (25 ng/ml) and iguratimod (0.5 *μ*g/ml) for 24 h. Apoptosis was determined by flow cytometry. Typical flow plots were shown. The data were shown as the mean ± SD for three independent experiments. The data were analyzed using one-way ANOVA followed by Turkey's test. ^∗^*P* < 0.05, ^∗∗^*P* < 0.01, ^∗∗∗^*P* < 0.001 versus the DMSO or control group.

As we know, RA-FLSs are relatively resistant to apoptosis with a prolonged growth characteristic. Therefore, we investigated whether iguratimod promoted apoptosis of RA-FLSs. RA-FLSs were treated with TNF-*α* (25 ng/ml) plus different concentrations of iguratimod. As shown in Figure 5, the early apoptotic cells (PI negative, Annexin V positive) and late apoptotic or dead cells (PI positive, Annexin V positive) were both markedly increased after treatment with iguratimod. The upregulation trend of apoptosis was paralleled with the concentration of iguratimod: higher concentration led to a higher apoptosis rate. We further found that proapoptotic caspase 3 mRNA was increased, while cellular FLICE-like inhibitory protein (cFLIP), an inhibitor of apoptosis, was reduced in TNF-*α*-treated RA-FLSs (Figures 5(d) and 5(e)). It was reported that pan-caspase inhibitors such as z-VAD-fmk or ROS inhibitor called N-acetyl-l-cysteine (NAC) can reverse apoptosis [22]. We found that z-VAD-fmk can reverse iguratimod-induced apoptosis in RA-FLSs (Figure 5(f)). NAC can very slightly reduce the iguratimod-induced apoptosis in RA-FLSs, but there was no significant difference (Figure 5(f)). The findings suggest that iguratimod promote TNF-*α*-induced early and late apoptosis. The iguratimod-induced apoptosis depended on the caspase-dependent pathway.

### 3.6. Iguratimod Directly Interacts with JNK and P38 MAPK

**Figure 6:**
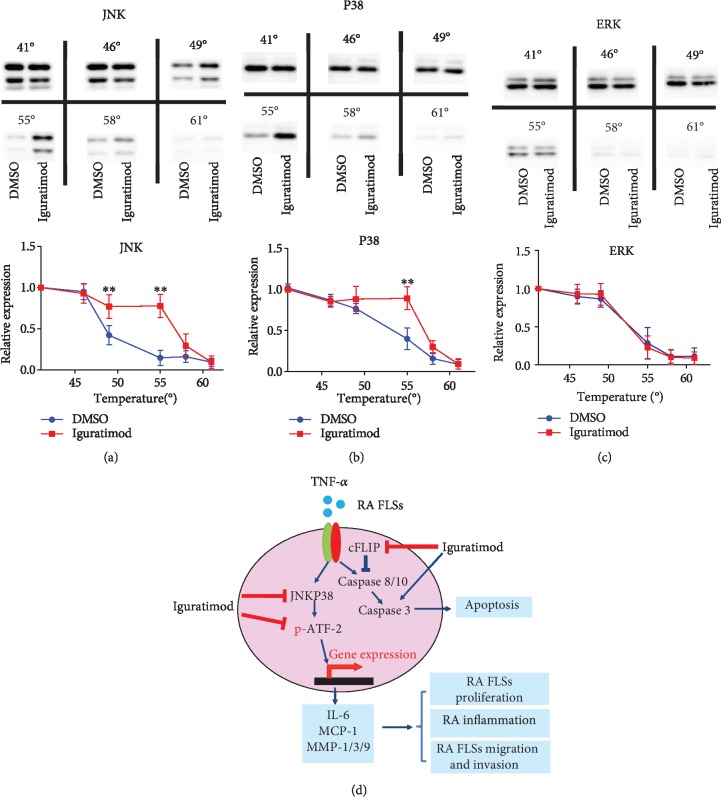
The possible mechanisms for interacting with RA-FLSs by iguratimod. (a–c) Cellular thermal shift assay (CETSA) of RA-FLSs with or without iguratimod treatment was assessed. The expression of JNK, P38, and ERK proteins were measured at different temperatures. The relative protein expression at the indicated temperature was calculated based on the band density of the related protein at 41°. The experiments were performed at three independent occasions (totally 5 RA-FLSs lines). ^∗∗^*P* < 0.01 versus DMSO group. (d) The possible mechanisms by which iguratimod targets the aggressive behavior and apoptosis of RA-FLSs.

In order to elucidate the mechanism by which iguratimod affect the RA-FLSs, we did a cellular thermal shift assay which has been extensively applied to purified proteins in the drug discovery industry and in academia to detect biological interactions. The assay involves treatment of cells with a compound of interest, heating to denature and precipitate proteins, cell lysis, and the separation of cell debris and aggregates from the soluble protein fraction. Whereas unbound proteins denature and precipitate at elevated temperatures, ligand-bound proteins remain in solution. We observed the presence of the protein on the western blots at the lower temperatures followed by its disappearance as the temperature increased. Then, we found that protein JNK and P38 bands were still apparent in the iguratimod-treated group, but were very weak in the DMSO group at 55°C. The expression of ERK protein was always the same in either the iguratimod-treated group or the DMSO group at different temperatures. These results suggest that iguratimod can directly interact with JNK and P38 protein, but not with ERK protein (Figures 6(a)–6(c)).

Finally, the possible mechanisms by which iguratimod targets the aggressive behavior and apoptosis of RA-FLSs is shown schematically, as a summary of the described results (Figure 6(d)).

## 4. Discussion

As it has been shown before, hyperplasia of RA-FLSs contributes to chronic inflammation and joint destruction in RA [2]. Here, we clearly demonstrated that iguratimod significantly reduced the proliferation of RA-FLSs in a dose-dependent manner *in vitro* as measured by Edu and CCK-8 tests. We also found that iguratimod significantly inhibited migratory and invasive capacities of RA-FLSs in a dose-dependent manner *in vitro* by the wound test and transwell assay. The inhibitory effect of iguratimod on invasiveness and MMP-1 and MMP-3 production of RA-FLSs has been reported before [12, 13]. Our results not only confirmed these previously published results [12, 13] but also found that MMP-9, IL-6, and MCP-1 expressions were all decreased in RA-FLSs after iguratimod treatment. Indeed, TNF-*α* and MCP-1 are potent inflammatory cytokines involved in the pathogenesis of RA [23, 24]. Interestingly, MCP-1 is a central chemokine, produced by monocytes and macrophages in the inflammatory response. MCP-1 expression was higher in RA patients compared with controls, in particular among anticitrullinated protein antibody-positive RA patients, in samples drawn prior to the onset of RA [25]. This study indicated that iguratimod targeted MMPs and potent inflammatory cytokines by inhibiting their production in RA-FLSs.

Most importantly, RA-FLSs were protected from apoptosis partly due to strong survival signaling [2]. Increased expression of several antiapoptotic proteins was proposed to explain the dysregulation of apoptosis and accumulation of FLSs in RA synovium [26]. For instance, TNF-*α* induced increase in cFLIP expression protected RA-FLSs from apoptosis [26]. Here, we reported for the first time that iguratimod promoted the apoptosis of RA-FLSs. The early apoptotic cells and late-stage apoptotic cells were both increased after treatment with iguratimod, suggesting that iguratimod promotes TNF-*α*-induced early- and late-stage apoptosis. We also found that proapoptotic caspase 3 mRNA level was increased, while antiapoptotic cFLIP mRNA expression was reduced. Furthermore, z-VAD-fmk but not NAC can reverse iguratimod-induced apoptosis in RA-FLSs. Therefore, iguratimod could act on RA-FLSs through the direct trigger of the apoptotic pathway. This effect may be due to an increase in proapoptotic caspase 3 expression, a decrease in cFLIP expression, and caspase-dependent pathway.

The MAPK signaling pathway, including JNK, P38, and ERK, is responsible for regulating a variety of cellular activities including proliferation, differentiation, survival, and death [27]. It has been reported that JNK, P38, and ERK are highly activated in RA-FLSs [2, 28, 29]. IL-6-induced ERK signaling was inhibited by iguratimod [12, 13]. Furthermore, IL-17-induced JNK, P38, and ERK signaling were inhibited by iguratimod [4]. Distinct from the above two studies, our results indicated that iguratimod treatment reduced the TNF-*α*-induced JNK and P38 signaling, but only slightly affected ERK signaling. In addition, a thermal shift assay showed that iguratimod can directly interact with JNK and P38 protein. Furthermore, we demonstrated that the ATF-2, the downstream protein of JNK and P38 signaling pathway, was suppressed by iguratimod. It had been reported that ATF-2 can be induced by TNF-*α* in RA-FLSs, contributing to the pathogenesis of RA [21]. It suggested that iguratimod can treat RA partly acting on JNK/P38-ATF-2-mediated pathway.

## 5. Conclusions

We clearly demonstrated that iguratimod treatment significantly reduced the proliferation, migration, and invasive capacities of RA-FLSs in a dose-dependent manner. Iguratimod also suppressed MMPs and inflammatory cytokine production in RA-FLSs. Moreover, iguratimod increased the apoptosis of RA-FLSs. Due to its contribution to altering the aggressive behavior and apoptosis of RA-FLSs, our results suggest that iguratimod has properties to make it an effective drug for multitarget therapeutic approaches to RA.

## Data Availability

The statistically analyzed data used to support the findings of this study are included within the article. The raw data used to support the findings of this study are available from Weiqian Chen, the corresponding author, upon request.
